# Prophylactic ondansetron for preventing intraoperative shivering, nausea and vomiting during spinal anesthesia for cesarean section: a randomized controlled trial

**DOI:** 10.3389/fphar.2024.1500642

**Published:** 2024-12-10

**Authors:** Yuan Zhang, Fen Xia, Wangping Zhang, Anqing Lv

**Affiliations:** ^1^ Department of Anesthesiology, Shaoxing People’s Hospital, Shaoxing Hospital of Zhejiang University, Shaoxing, China; ^2^ Department of anesthesiology, Jiaxing Women and Children’s Hospital, Jiaxing, China

**Keywords:** ondansetron, shivering, nausea and vomiting, spinal anesthesia, cesarean section

## Abstract

**Background:**

Shivering, nausea and vomiting are common complications in cesarean section during neuraxial anesthesia. The aim of this study was to investigate the effects of prophylactic use of ondansetron on intraoperative shivering, nausea and vomiting in women undergoing cesarean section.

**Methods:**

A total of 80 full-term parturients were randomly divided into the ondansetron group and the control group. The ondansetron group received 0.075 mg/kg of ondansetron 15 min before anesthesia, the control group were given the same volume of saline solution. The incidence of shivering, nausea and vomiting was noted. The occurrence and severity of shivering and other outcomes, such as hypotension, bradycardia and dizziness were recorded during the surgery. Umbilical arterial blood was analyzed, and the neonatal Apgar scores were assessed.

**Results:**

The incidence of grade ≥1 shivering was lower in ondansetron group. The incidence of shivering, nausea and vomiting was significantly lower in ondansetron group than the control group (2.5% vs. 22.3%, *P* = 0.007, 2.5% vs. 40%, *P* = 0.001, respectively). No significant differences were observed in the incidence of hypotension, bradycardia, headache and dizziness between the 2 groups (*P* > 0.05). The umbilical artery pH and neonatal Apgar score were similar between the 2 groups (*P* > 0.05).

**Conclusion:**

Prophylactic use of ondansetron could prevent intra-operative shivering and reduce the incidence of nausea and vomiting in cesarean section under spinal anesthesia without increasing the incidence of maternal and infant adverse events.

**Clinical Trial Registration:**

https://www.chictr.org.cn, identifier ChiCTR2100042453

## 1 Introduction

Shivering is a common intraoperative complication in cesarean section under neuraxial anesthesia (Abdel-Ghaffar and Moeen, 2019). The incidence of shivering was 42.0% in patients after neuraxial anesthesia for cesarean section (Qi et al., 2022). It causes an unpleasant feeling, increases oxygen consumption and leads to adverse events, such as surgical site bleeding, cardiac events, wound infections, raised lactic acidosis and carbon dioxide production (Frank et al., 1995; Capogna and Celleno, 1994). It is crucial to prevent and treat shivering effectively in patients undergoing caesarean section.

Literature reported that midazolam, propofol, ketamine, sufentanil, dexmedetomidine, magnesium and tramadol may treat postoperative shivering or decrease the incidence of postoperative shivering (Jung et al., 2018; Nakayama et al., 2021; Honarmand and Safavi, 2008; Solhpour et al., 2016; Singh et al., 1994; Heid et al., 2008).

Ondansetron is a highly selective serotonin receptor antagonist. It is widely used for preventing and treating all kinds of nausea and vomiting. Some studies found that intravenous ondansetron could reduce the drop of blood pressure in the cesarean section because it can also suppress the serotonin-induced vasodilation by reducing Bezold-Jarisch reflex (Owczuk et al., 2008; Wang et al., 2014). Moreover, literature reported that intravenous ondansetron could effectively decrease the occurrence of supine hypotension syndrome after spinal anesthesia (Zhang et al., 2024). However, the study on prophylactic use of ondansetron for intra-operative shivering, nausea and vomiting is few in cesarean section under spinal anesthesia. This study investigated the effects of prophylactic use of ondansetron on intraoperative shivering, nausea and vomiting during cesarean section when ondansetron administration prior to spinal anesthesia.

## 2 Methods

This study was conducted in accordance with the Declaration of Helsinki. This trial was registered in the Chinese Clinical Trials Registry (registration number: ChiCTR2100042453). Parturients were randomly divided into the ondansetron group and the control group. Randomization was carried out by opening an opaque, sealed envelope containing a sequential number. The allocation sequence was generated using random permuted block randomization. The study medications were prepared by a nurse who was not involved in this study. The surgeons, anesthetists and investigators were blinded to the group allocation.

### 2.1 Study objects

After approval the Hospital Ethical Committee and written informed consent, a total of 80 parturients undergoing elective cesarean section were enrolled in this study. Inclusion criteria were as follows: American Society of Anesthesiologists physical status II, singleton gestation ≥37 weeks, height 151–175 cm and weight 50–90 kg. Exclusion criteria: the patients with pregnancy-related hypertensive disease, cerebrovascular disease, anticholinergics, sedative drugs and any contraindications to the use of spinal anesthesia.

All paturients have no premedications and were fasted for 8 h. The temperature of the operating room was set at 23°C. On arrival in the operating room. The electrocardiogram, noninvasive blood pressure (BP), pulse oxygen saturation (SpO_2_) and heart rate (HR) were measured using anesthesia monitor. The BP and HR were recorded at 1 min intervals until baby delivery, then recorded at 5 min intervals. Subsequently, an 18-G intravenous catheter was inserted into the forearm vein. Lactate Ringer’s solution was infused at a rate of 10 mL. kg^−1^ .h^−1^ until the end of surgery.

The ondansetron group were given 0.075 mg/kg of ondansetron via intravenous injection 15 min before spinal anesthesia, and the control group were given the same volume of 0.9% normal saline. The study drugs were prepared by nurses who were not involved in this study. Anesthesia puncture was performed in the left lateral position. An 18-G Tuohy needle was introduced into the epidural space at the estimated L_3_-L_4_ vertebral interspace and a 25-G spinal needle was inserted into the subarachnoid space via the Tuohy needle. After free flow of cerebrospinal fluid, a mixture of 0.5% hyperbaric bupivacaine 10 mg + 10% glucose (total 2 mL) was injected intrathecally over 10 s with the needle orifice facing cephalad, and an epidural catheter was inserted 4 cm cephalad into epidural space. Then patients were immediately turned to the supine position with a 10-degree tilt to the left side. Surgical incision was permitted after bilateral T6 sensory block of pain was achieved. Umbilical artery pH was measured immediately after delivery, and 10 U of oxytocin was injected muscularly *in utero*, then 5 U of oxytocin was continuously infused till the end of surgery.

### 2.2 Measurement

The assessment of shivering was performed using a four-point rating scale (0 = none, 1 = mild fasciculation at a single muscle group, 2 = fasciculation at more than one muscle groups and 3 = strong activity of the entire body). Shivering was defined as scores of 1 or more.

The neonatal outcomes including the neonatal Apgar scores at 1 and 5 min and umbilical artery pH were assessed. The incidence of maternal shivering, hypotension, bradycardia, headache, dizziness, nausea and vomiting were recorded. Hypotension was defined as SBP <80% of baseline and was treated with 50 µg phenylephrine by intravenous injection. Bradycardia was defined as HR < 60 beats/min and was treated with 0.3–0.5 mg atropine by intravenous injection. The maximal level of sensory block was measured at 10 min of intrathecal injection using a pinprick at 2-min intervals.

### 2.3 Statistical analysis

The primary outcome of this study was the incidence of shivering. The secondary outcome was the incidence of nausea and vomiting. A pilot study with 10 patients in each group showed that the incidence of shivering was 20% and 0, respectively. The incidence of shivering was reduced by 20% in the ondansetron group. A sample size of 36 patients was calculated using Power Analysis and Sample Size software 2020 (NCSS, LLC, Kaysville, UT, United States) to achieve an α of 0.05 and a power of 0.8. The sample size in each group was increased to 40 to allow for dropouts. The numerical variables were analyzed using a *t-*test for independent samples. The categorical data were compared using a Chi-squared test or Fisher’s exact test. A *P*-value of <0.05 was considered statistically significant.

## 3 Results

**FIGURE 1:**
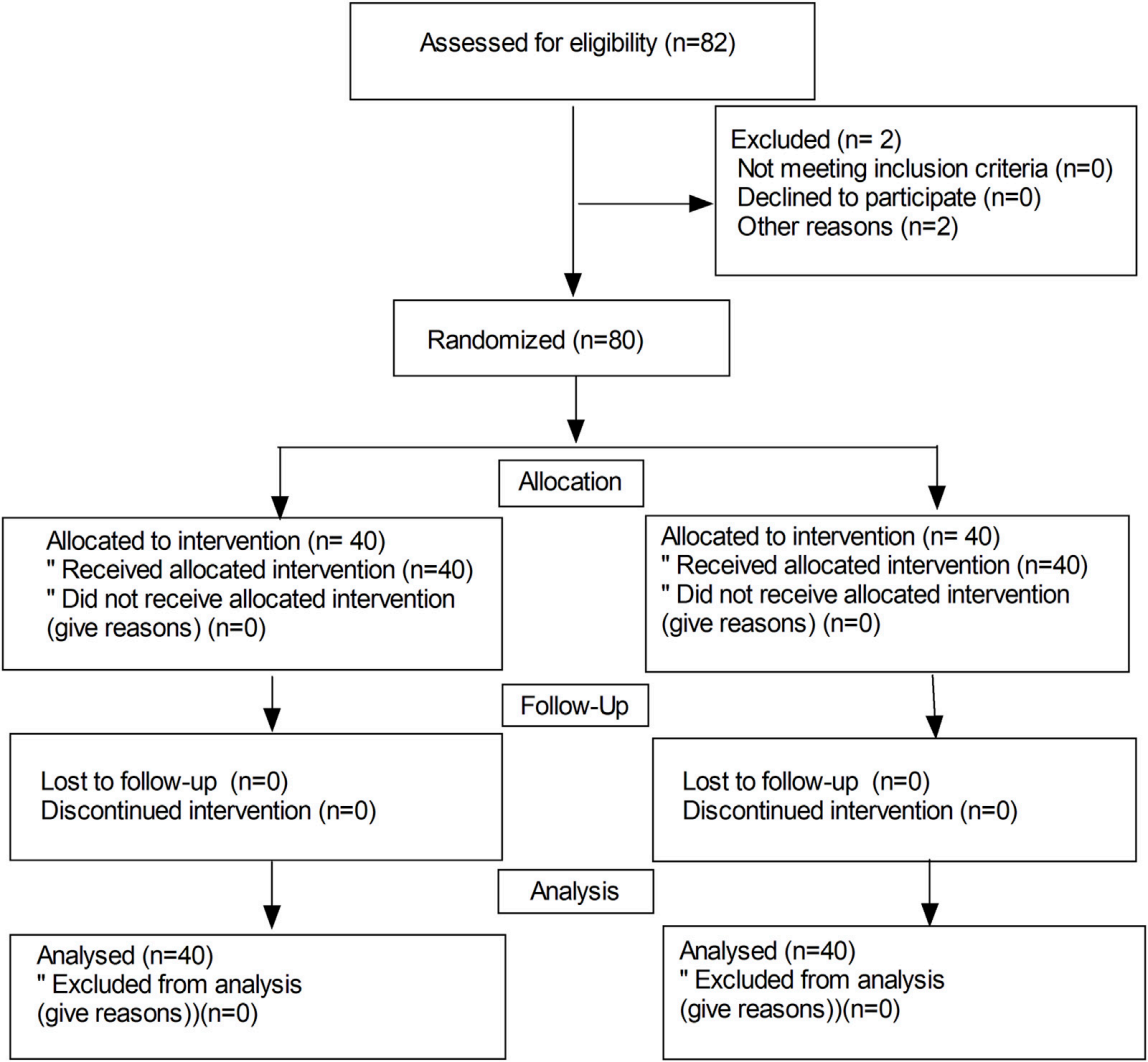
Flow diagram of the study.

Figure 1 shows the flow diagram of the study. Eighty-two parturients were recruited and 80 parturients completed the study. No differences were observed in terms of the age, weight, height, body mass index, and gestational week between the 2 groups (Table 1). There were no significant differences in the duration of surgery, blood loss, total amount of fluid, maximal levels of sensory block, delivery time of baby, and vasoconstrictor use between the 2 groups (*P* > 0.05).

**TABLE 1:** Characteristics of the parturients.

Index	Ondansetron group (*n* = 40)	Control group (*n* = 40)	*P*-value
Age (year)	30.1 ± 3.5	30.6 ± 3.4	0.516
Height (cm)	160.2 ± 5.1	161.1 ± 4.9	0.414
Weight (kg)	71.1 ± 9.4	73.9 ± 9.4	0.180
Body mass index (kg/m^2^)	27.3 ± 2.8	27.1 ± 3.1	0.202
Gestational age (week)	38.6 ± 0.9	38.5 ± 1.0	0.642

Data are presented as mean ± SD.

**FIGURE 2:**
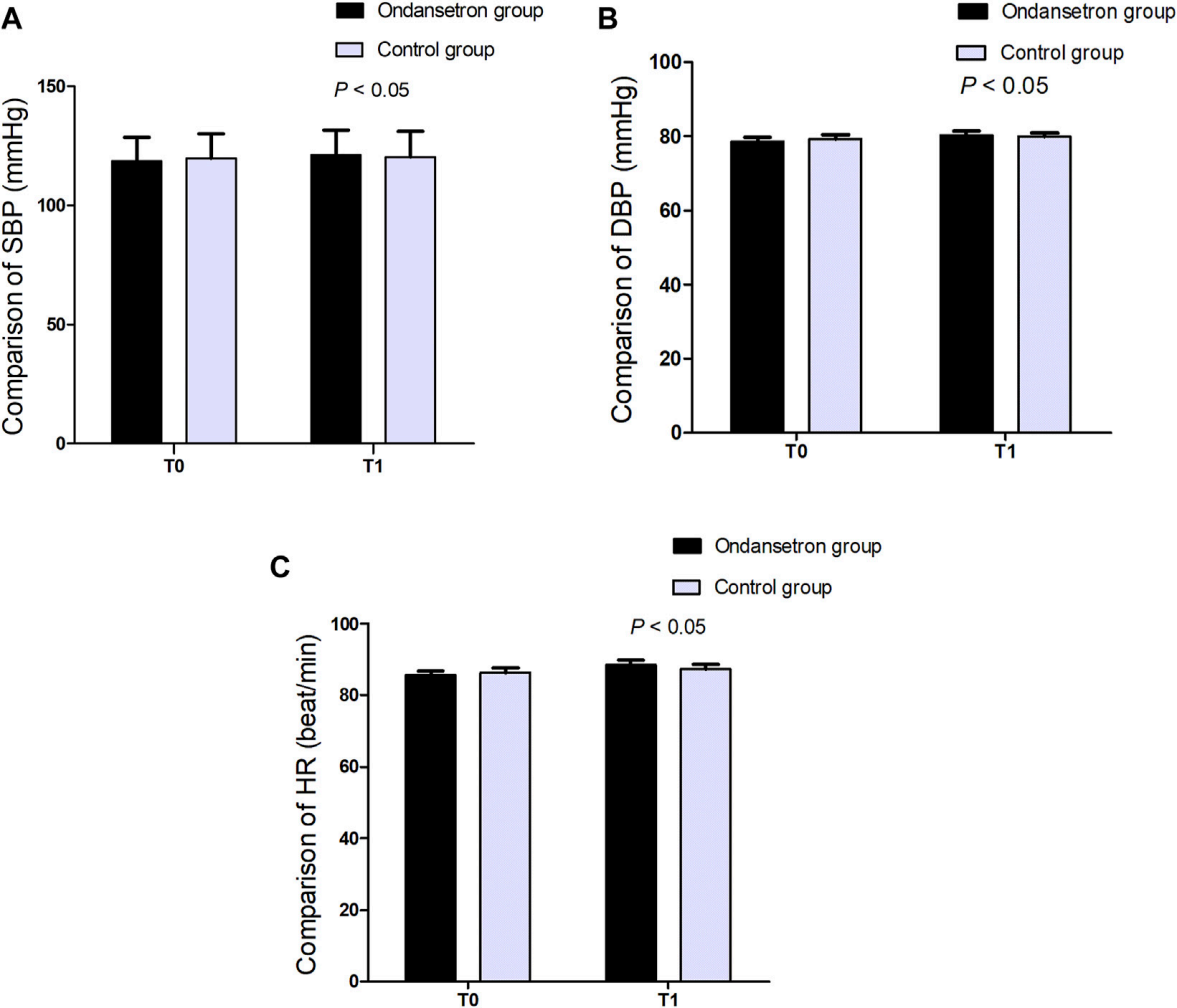
**(A–C)**. Comparison of SBP, DBP, and HR between the two groups, *P* > 0.05. T0: baseline value, T1: 15 min after administration.

Blood pressure and heart rates after drug administration were slightly increased in the ondansetron group, but no significant differences were observed between the 2 groups (in Figure 2).

The incidence of shivering, nausea and vomiting was lower in the ondansetron group than in the control group (2.5% vs. 22.3%, *P* = 0.007, 2.5% vs. 40%, *P* = 0.001, respectively), while no statistical significances were observed between the 2 groups in terms of hypotension, bradycardia, headache, dizziness and vasoconstrictor use (*P* > 0.05) (in Table 2). The neonatal 1-min Apgar score and 5-min Apgar score were similar between the 2 groups (in Table 2), and there were no statistical significances in umbilical arterial pH between the 2 groups (*P* > 0.05).

**TABLE 2:** Maternal and neonatal outcomes.

Index	Ondansetron group (*n* = 40)	Control group (*n* = 40)	*P*-value
Duration of surgery (min)	33.1 ± 6.6	34.4 ± 7.5	0.384
Blood loss (mL)	212 ± 28	218 ± 36	0.409
Total amount of fluid (mL)	742.3 ± 49.9	744.5 ± 62.8	0.860
Maximal levels of sensory block [T4/T6] (n)	10/30	9/31	0.793
Delivery time (min)	17.5 ± 2.5	16.8 ± 2.6	0.224
Shivering (n)	1 (2.5)	10 (22.5)	0.007
Nausea and vomiting (n)	1 (2.5)	12 (30)	0.001^a^
Hypotension (n)	14 (35)	16 (40)	0.644
Bradycardia (n)	1 (2.5)	2 (5)	0.999
Dizziness (n)	0 (0)	0 (0)	0.999
Headache (n)	0 (0)	0 (0)	0.999
Vasoconstrictor use (n)	14 (35)	16 (40)	0.644
1-min Apgar score	9.1 ± 0.7	9.0 ± 0.6	0.581
5- min Apgar score	9.5 ± 0.5	9.6 ± 0.5	0.508
Umbilical artery pH	7.32 ± 0.06	7.31 ± 0.05	0.423

Data are presented as mean ± SD, or numbers (percentage).

^a^ *P* < 0.05.

Shivering scores are showed in Table 3. There was only one case of shivering (shivering scores of 1) in ondansetron group. While there were ten cases of shivering (shivering scores of ≥1) in ondansetron group. The incidence of shivering scores of 0 was higher in ondansetron group compare to the control group (*P*= 0.007), while the incidence of grade ≥1 shivering was lower in ondansetron group (*P*= 0.007).

**TABLE 3:** Shivering scores during study period.

Index	Ondansetron group (*n* = 40)	Control group (*n* = 40)	*P*-value
Shivering score = 0	39	31	0.007^a^
Shivering score = 1	1	5	0.201
Shivering score = 2	0	2	0.494
Shivering scores = 3	0	2	0.494

Data are presented as numbers.

^a^ *P* < 0.05.0 = none, 1 = mild fasciculation at a single muscle group, 2 = fasciculation at more than one muscle groups and 3 = strong activity of the entire body). Shivering was defined as scores of 1 or more.

## 4 Discussion

In this study, we found that prophylactic use of ondansetron could prevent intra-operative shivering and reduce the incidence of nausea and vomiting in cesarean section under spinal anesthesia without increasing the incidence of maternal and infant adverse events.

The exact etiology of shivering is unknown, and its treatment is also not well-defined (Zhang et al., 2024; Subramani et al., 2020). The occurrence of shivering is related to many factors during cesarean sections. The previous studies suggested that anxiety, hypotension, and hypothermia may be associated with intraoperative shivering during cesarean sections (Frank et al., 1995; Wódarski et al., 2020).

Literature reported that most of pregnant women experienced anxiety on the day of the cesarean section (Kuo et al., 2014), and anxiety could contribute to shivering during cesarean sections (Wódarski et al., 2020). In our study, we found that ondansetron could obviously decrease the incidence of intra-operative shivering in cesarean section. The mechanism of ondansetron for preventing intra-operative shivering is likely as follows: antagonists of serotonin receptor inhibit the neurotransmission involved in thermoregulation in the hypothalamus (Sharma et al., 2021). Voronova (2021) found that serotonin receptors regulated body temperature in warm-blooded animals, and caused a decrease in body temperature by activation of serotonin receptors. Suchacki et al. (2023) also found that serotonin regulated body temperature by suppressing human brown adipose tissue activation and inhibiting the serotonin transporter.

Liu et al. (2020), Suchacki et al. (2023) found that ondansetron could decrease the incidence of shivering after spinal anesthesia for urgent cesarean section without dizziness. Lakhe et al. (2017), Liu et al. (2020) also found that prophylactic use of ondansetron was effective in preventing shivering post spinal anesthesia without untoward effects. Their findings were consistent with our results.

Nausea and vomiting affects up to 80% of pregnant women worldwide (Lakhe et al., 2017). The symptoms of nausea and vomiting of pregnancy vary in severity ranging (Matthews et al., 2014). In the present study, we also shown that ondansetron could decrease the incidence of intra-operative nausea and vomiting in cesarean section. Ondansetron is a selective serotonin receptor antagonist, which produces antiemetic effect by blocking 5-HT_3_ receptor or inhibiting the emetic center.

In our study, blood pressure and heart rate after drug administration were slightly increased in the ondansetron group, but no significant differences were observed between the 2 groups. It is well known that antagonists of 5-HT_3_ receptor (ondansetron) can suppress the serotonin-induced vasodilation by reducing Bezold-Jarisch reflex. Ondansetron did not cause an obvious increase in blood pressure or heart rate when ondansetron administration prior to spinal anesthesia. This is because a drop in blood pressure can trigger the Bezold-Jarisch reflex in the case of hypotension. That is to say, when blood pressure is within the normal range, the Bezold-Jarisch reflex will not occur. The incidence of hypotension and bradycardia was similar between the 2 groups. However, some studies found that intravenous ondansetron could reduce the drop of blood pressure during spinal anesthesia for cesarean section by reducing Bezold-Jarisch reflex (Owczuk et al., 2008; Wang et al., 2014). This could be due to the small sample size.

In this study, no differences were observed in umbilical artery pH and Apgar scores of the neonates between the 2 groups. It indicated that ondansetron had no adverse effects on the neonates. Umbilical artery pH is a sensitive parameter for diagnosing fetal asphyxia. It is mainly associated with fetal perfusion. As we actively treated the hypotension when hypotension occurred, no fetal asphyxia was observed. Ondansetron attenuates the spinal anesthesia-induced hypotension by inhibiting the Bezold-Jarisch reflex and reducing serotonin-induced vasodilation, as is beneficial to perfusion of fetus. The safety of ondansetron use is controversial in pregnancy. Zambelli-Weiner and colleagues found that ondansetron exposure in the first trimester was associated with an increased risk of heart defects (adjusted odds ratio [OR]: 1.52, 95% confidence interval [CI]: 1.35–1.70) and with a non-significant trend toward oral clefts (OR: 1.32, 95% CI 0.76–2.28) (Slattery et al., 2022). Huybrechts and colleagues found an increased risk of oral clefts (adjusted relative risk [RR] 1.24, 95% CI 1.03 to 1.48; 3 additional cases per 10,000 women treated in the 1st trimester), but not of heart defects (RR: 0.99, 95% CI 0.93–1.06) (Zambelli‐Weiner et al., 2019). However, Larrimer, et al. (2017), Huybrechts et al. (2018) found that ondansetron use in pregnancy had no clinically significant adverse neurobehavioral effects or obstetrical outcomes.

### 4.1 Limitations

Since anxiety can be a factor in shivering, preoperative anxiety may influence the results. Moreover, environmental temperature can affect the accuracy of research results. Finally, the safety of ondansetron in the neonates needs further research.

## 5 Conclusion

Prophylactic use of ondansetron could prevent intra-operative shivering and reduce the incidence of nausea and vomiting in cesarean section under spinal anesthesia without increasing the incidence of maternal and infant adverse events.

## Data Availability

The raw data supporting the conclusions of this article will be made available by the authors, without undue reservation.
